# Surface topological differences of phage infected uropathogenic *Escherichia coli* (UPEC) strains, revealed by atomic force microscopy

**DOI:** 10.1186/s40064-016-3781-1

**Published:** 2016-12-29

**Authors:** Bassamah Hanif, Nusrat Jamil, Muhammad Raza Shah

**Affiliations:** 1 Department of Microbiology, University of Karachi, Karachi, Pakistan; 2 International Centre for Chemical and Biological Sciences (ICCBS), University of Karachi, Karachi, Pakistan

**Keywords:** UPEC, AFM, Short latent period, Multiple internal projections, Single site, Central depression

## Abstract

**Background:**

Atomic force microscopy (AFM) is an advance microscopic technique that provides three dimensional structures of cell surfaces with high resolution. In the present study AFM was used for comparative analysis of surface topology of phage infected and uninfected Uropathogenic *Escherichia coli* (UPEC) cells. Two UPEC strains NE and HN were isolated from urine samples of Urinary tract infection patients and their specific narrow host range lytic phages 3S and HNΦ were isolated from the sewage of different areas.

**Results:**

On the basis of one step growth curve both phages characterized as short latent period phages with latency period of about 30 min. On AFM analysis significant difference in topology of healthy and infected cells were observed. It was hypothesized that progeny of both lytic phages released out from their respective host cells in different manner. The image of 3S infected UPEC host cells (NE) revealed multiple internal projections which showed progeny phages released out from host cells through these multiple sites. Whereas images of HNΦ infected HN host cells showed central depression which illustrated that new phages released out through single exit point from the middle of cell.

**Conclusions:**

These results are significant to extend future studies on isolated phages as an effective tool for phage therapy.

## Background

*Escherichia coli* (*E. coli*) is the most common causative agent of urinary tract infections (UTI) that if not treated properly can lead to kidney failure. The drug of choice for treating UTI includes Sulfamethoxazole–trimethoprim and fluoroquinolones (Hooton 2012). However, the treatment of UTI is becoming much difficult because Uropathogenic *E. coli* strain (UPEC) shows resistance against these antibiotics. More importantly, the incomplete courses of antibiotics adversely affect the normal gut flora and micro flora found in periurethral area of women. This adverse effect on normal flora enhances resistance of uropathogens against these antibiotics and increases the chances of recurrences of UTI. Therefore, there is an emerging trend towards alternative treatment of UTI that includes phage therapy, effects of functional food products, probiotics, and vaccines (Foxman and Buxton 2013).

Previous studies has shown that efficacy of particular phage in phage therapy depends on different parameters which has to be determined to employ for phage therapy. These properties of antibacterial substances are broadly classified as efficient pharmacodynamics (PD) and pharmacokinetics (PK). For phage, antibacterial properties depend on its binding to host cells, shorter generation time and latent period with large burst size (Drulis-Kawa et al. 2012). These characteristics of the phage can be studied by single step growth curve of phage, in which phage binding to host cell, latent period, and burst size can be calculated. With microbiological methods, effect of phage on host cells can be visualized by different techniques like electron microscopy or more advance technique Atomic force microscopy (AFM) can also be deployed. AFM is the latest microscopic technique that provides three-dimensional structure of sample surfaces with high resolution in the range of nanometre (Müller and Dufrêne 2011). The superiority of this technique over other methods includes affirmation regarding the physical properties of cell at single cell level without any cell manipulation and living cell surface imaging at high resolution in absence of vacuum conditions (Dufrêne 2002). Many researchers are using AFM for studying different morphologies of phages and their effect on the infected host. The interaction of *Acinetobacter baumannii* with its lytic phage was detected and observed by Dubrovin et al. (2012). They also reported that the phage has high adsorption rate and ability to disperse bacterial aggregates of *A. baumannii*, which is a nosocomial pathogen. Kuznetsov et al. (2013) have identified some unique tail appendages of marine bacteriophages of *Cyanobacteria synechococcus*. Dubrovin et al. (2008) characterized three different types of phages on the basis of AFM and Transmission Electron Microscopy (TEM) and studied the infection process of bacterial cells by bacteriophages using AFM.

In present study isolation of lytic phage against UPEC strain was carried out. These strains were characterized on the basis of host range, single step growth curve and finally uninfected and phage infected host cells are compared on basis of surface topological differences by Atomic force microscopy (AFM).

## Methods

### Isolation of host bacteria and lytic phage

Sewage samples from different localities of Karachi and urine samples of patients with UTI were collected in sterile amber bottles. Urine samples were filtered then sediment was streaked on Brain Heart Infusion agar (BHI) and MacConkey agar plates, incubated at 37 °C overnight for isolation of host *E. coli*. Next day, isolated colonies were Gram stained and identified by inoculating Triple sugar iron (TSI) slants and Eosin methylene blue agar (EMB) agar plates. The identified bacterial culture used as host and spot assay was performed on Luria-Bertani (LB) agar with 10 µl of filtered sewage samples on lawn of host cells, plates were incubated at 37 °C overnight. Next day, samples were selected for lysate preparation based on lysis of bacterial cells.

### Phage lysate preparation

Five milliliter of L.B. broth inoculated with host cells was incubated at 37 °C overnight. Next day, this 5 ml culture was added to 15 ml of fresh L.B. broth in sterile flask, incubated in shaking incubator for 2 h at 37 °C. Following the incubation, culture mixed with 80 ml of sterile sewage sample, 10 mM CaCl_2_ and incubated in shaker at 37 °C overnight. Next day, a few drops of chloroform were added in the mixture and vortex vigorously for 15 min, spun at 5000 rpm for 20 min and filtered through 0.45 µm Millipore membrane filter. The filtrate was collected in sterile amber bottles (Sundar et al. 2009).

### Amplification of plaque

Plaque assay was performed by mixing 100 µl of log phase host cells with 100 µl of phage lysate in 3 ml melted L.B. semisolid agar tube with 10 mM CaCl_2_, overlaid on L.B. agar plates. Next day plates were examined for the presence of plaques and morphology of plaques was observed. Well isolated single plaque was picked out in 1 ml PDB with sterile Pasteur pipette. This single plaque suspension then used as lysate to perform plaque assay with host culture. The procedure was repeated for three times and final suspension was prepared in 5 ml L.B. broth using 100 µl bacterial culture, incubated at 37 °C in shaker overnight. Next day, this suspension was centrifuged at 5000 rpm, filtered and stored at 4 °C for future analysis.

### Determination of host range of phage

The two isolated phages were evaluated for ability to lyse different strains of *E. coli*, *Pseudomonas aeruginosa*, *Staphylococcus aureus*, *Salmonella paratyphi A* and *Shigella dysenteriae* by the help of spot assay. Further it was confirmed by plaque assay with above-mentioned protocol.

### Preparation of high titer phage stock

Initially plaque assay was performed as described but next day 10 ml Phosphate Dilution Buffer (PDB) was added in a plate containing clear and high plaques count. The top semi solid agar was scrapped off by sterile spatula and plate was left at room temperature for 2 h with regular swirling. After 2 h, PDB was collected in large tubes, spun at 5000 rpm for 20 min then supernatant was collected and filtered through 0.45 µm Millipore membrane filter and stored at 4 °C (Carey-Smith et al. 2006; Merabishvili et al. 2009).

### Determination of phage titer

The filtered phage stock was diluted from 10^−2^ up to 10^−6^ (PFU/ml) in PDB then 100 µl of each stock dilution and 100 µl of young host culture were added in 3 ml L.B. soft agar with 10 mM CaCl_2_ separately. The mixture was overlaid on L.B. agar plate then incubated overnight at 37 °C. Next day, plaques were counted and used to calculate titer of phage stock by formula of PFU/ml (Mudgal et al. 2006).

### Single step growth curve of phage

For single step growth curve initially 5 ml log phase host cells were cultured in two parallel tubes. One tube was served as control but other was phage infected tube from which 100 µl aliquot was taken, treated with phage lysate at 0.1 MOI with CaCl_2_ (10 mM), incubated for 10 min at 37 °C, and then spun at 3000 rpm for 5 min. After centrifugation, pellet was suspended in 2 ml L.B. broth in tube and samples were taken from this phage infected tube in two sets at 0, 15, and 30 up till 75 min interval. One set was untreated but other set was treated with 1% (vol/vol) chloroform to determine total PFU/ml. Initially 100 µl sample was taken at 0 min then diluted tenfold up to 10^−5^ with 900 µl PDB after this plaque assay of 10^−3^–10^−5^ dilution was performed on L.B. agar plates, and tube was incubated at 37 °C in shaker. This procedure was repeated at intervals of 15 min until 75 min for both set of samples separately. Next day, no of plaques were counted to calculate free PFU/ml, total PFU/ml, log PFU/ml, burst size and latent period and a graph was plotted for time versus PFU/ml using Excel (Yang et al. 2010; Hsieh et al. 2011).

### Atomic force microscopy

For observation of topological changes occurred on bacteria after phage infection, atomic force microscopy was employed. Initially, mica surface was freshly cleaved, coated with 0.01% Poly-l-Lysine solution, and left for drying overnight. Next day, log phase culture of host bacteria was grown in 1 ml L.B. broth. Then aliquot of log phase culture of host bacteria was pelleted by centrifugation, pellet was washed in distilled water, spun again, and suspended in 1 ml de-ionized distilled water (Udomrat et al. 2009; Zhang et al. 2011; Bolshakova et al. 2004). Two fifty micro litres of this bacterial suspension was treated with 100 µl of sterile phage lysate and incubated at 37 °C in shaking incubator for 30 min. After incubation, 10 µl of phage infected host bacteria suspension was spotted on Poly-l-Lysine coated mica surface by placing one drop of this phage infected host bacteria suspension on parafilm then 0.01% Poly-l-Lysine coated mica surface was touched by this spot present on parafilm using forceps and allowed to stand for 15 min. This method was introduced to avoid any blurred images due to deposition of sample in more amounts. Control Poly-l-Lysine coated mica surface were also visualized by spotting one of them with bacterial suspension without adding phage and other control mica surface with sterile phage lysate without bacteria were allowed to stand for 15 min. Samples were placed in sample plate of AFM (Agilent 5500 technologies, AZ, USA) and imaged in ambient condition at room temperature. Topographical images of treated and untreated cells were acquired and operated at tapping mode using Silicon nitride soft triangle-shaped cantilever (Veeco, model MLCT-AUHW); 0.01 and 0.1 N/m nominal spring constant.

## Results

### Isolation of host bacteria and phage

Lactose positive colonies on MacConkey plates were selected for further analysis. On gram staining pink, coccobacilli short rods were selected whereas on the basis of EMB and TSI slants it was identified as *E. coli* and one strain named as **HN** other as **NE**. It was found susceptible to phage isolated from sewage on spot assay. Lytic activity of phage was confirmed by performing plaque assay where phage produced middle-sized, clear, uncountable plaques. The phage was named as **HN Φ** for **HN** and **3S** for **NE**.

### Host range of isolated phage

For determination of host range of phages 3S and HN Φ different *E. coli*, *P. aeruginosa*, *S. aureus*, *S. dysenteriae, S. paratyphi A* strains were used but it was found lytic only on their host *E. coli* cells. Results for host range determination of these two phages are shown in the Table 1.

**Table 1:** Lytic pattern of 3S and HNΦ on propagating *E. coli* strains

Strains	Samples					
	Sewage 1		Sewage 2		Urine sample	
	Spot assay	Plaque assay	Spot assay	Plaque assay	Spot assay	Plaque assay
*Escherichia coli* (HN)	+++	+++	−	−	−	−
*Escherichia coli* (N.E)	−	−	+++	+++	−	−
*Escherichia coli* (E1)	−	−	−	−	−	−
*Escherichia coli* (E.Cu)	−	−	−	−	−	−
*Escherichia coli* (E.Ph)	−	−	−	−	−	−
*Pseudomonas aeruginosa* (P5)	−	−	−	−	−	−
*Pseudomonas aeruginosa* (P6)	−	−	−	−	−	−
*Staphylococcus aureus* (Hm)	−	−	−	−	−	−
*Staphylococcus aureus* (P.st)	−	−	−	−	−	−
*Staphylococcus aureus* (S.S)	−	−	−	−	−	−
*Salmonella paratyphi A*	−	−	−	−	−	−
*Shigella dysenteriae*	−	−	−	−	−	−

Key: **−** means no lysis, +++ means lysis

### Single step growth curve of phage

**Fig. 1:**
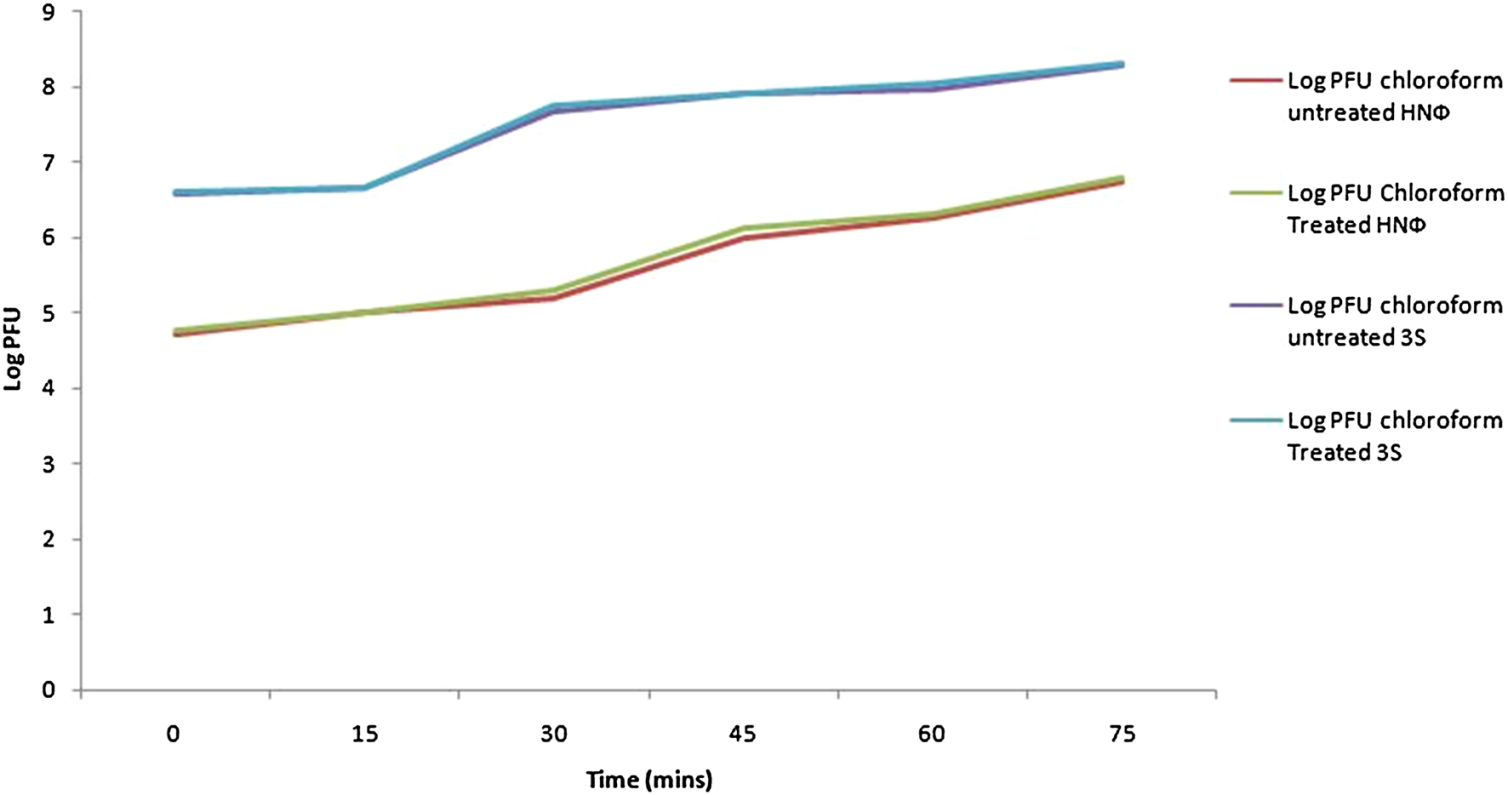
One-step growth curve of phage HNΦ and 3S

The titer of HN Φ phage stock was found to be 4.8 × 10^8^ PFU/ml and for 3S it was 7.1 × 10^10^ PFU/ml. The one step growth curve for both phages was determined by the method of Yang et al. (2010) and Hsieh et al. (2011). The burst size of HNΦ was 110 average progeny phage per infected cell and for 3S it was 42 average progeny phage. According to the graphical presentation latent period for HNΦ and 3S was about 30 min as shown in Fig. 1.

### Atomic force microscopy

**Fig. 2:**
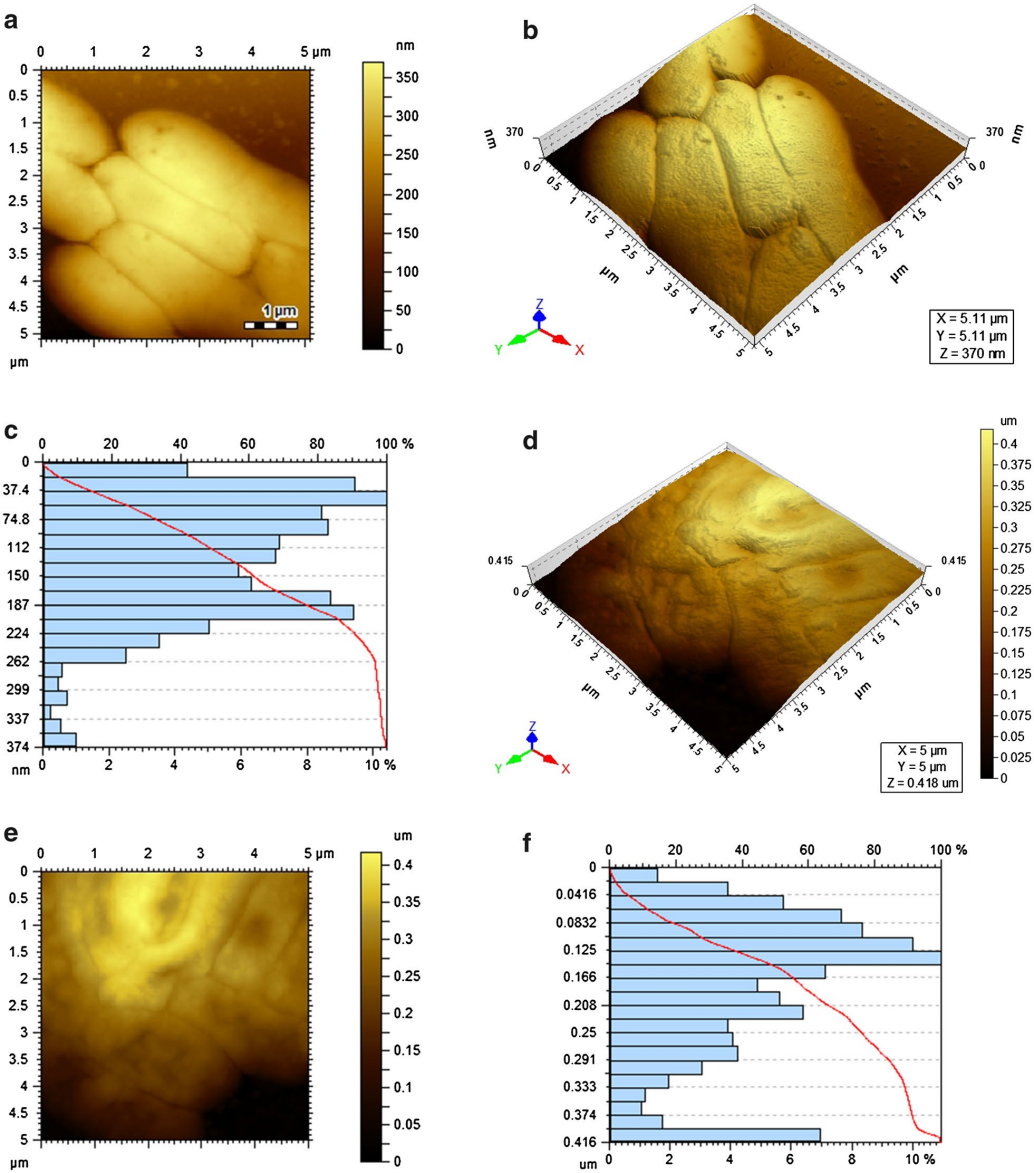
AFM images of HN control cells and HNΦ phage infected HN host cells on PLL coated grids

**Fig. 3:**
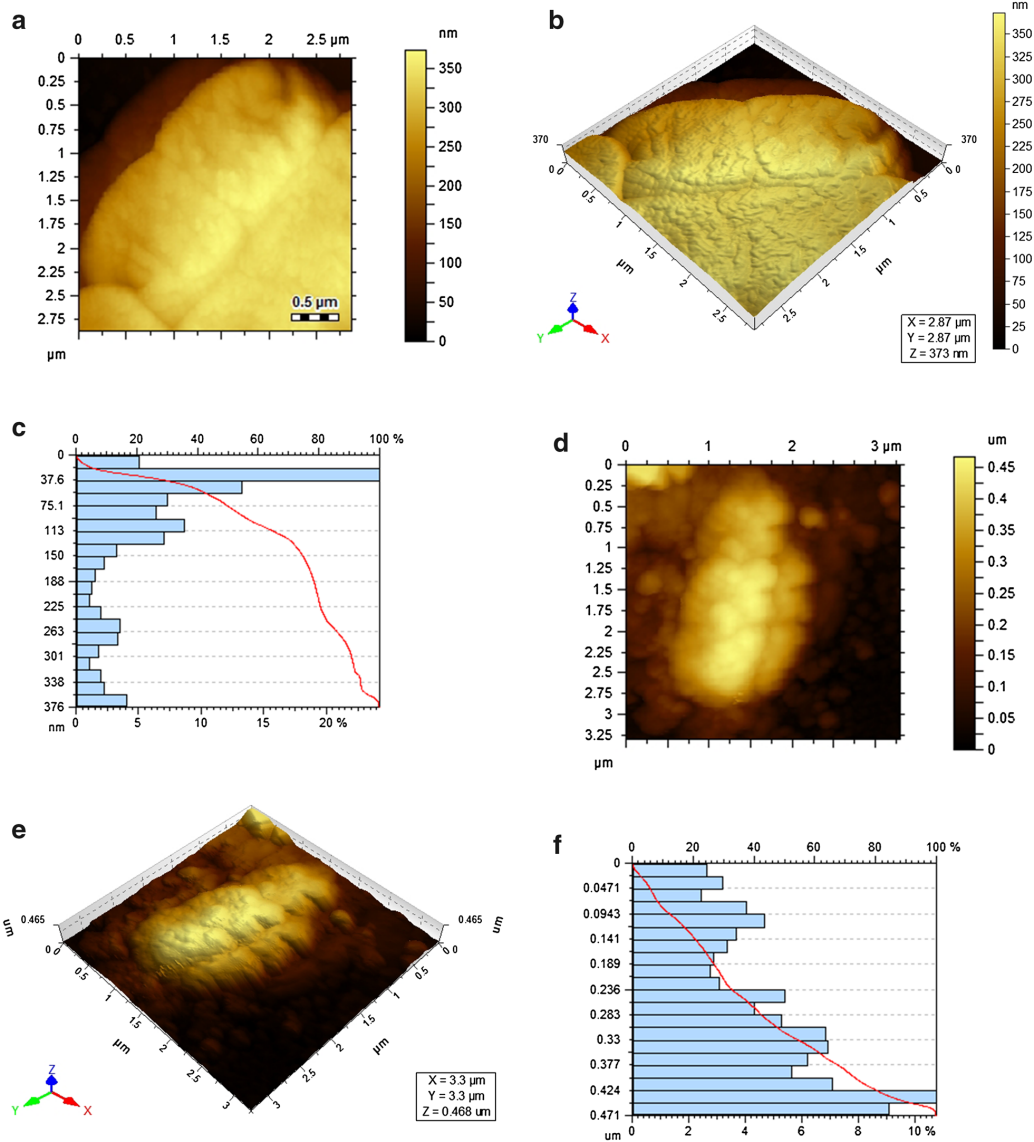
AFM images of NE control cells and 3S phage infected NE host cells on PLL coated grids

Significant difference between the surface topology of control and phage infected *E. coli* were observed as revealed by AFM analysis, host cells remained intact while phage-infected cells were disrupted. Moreover, host (NE) cells infected with 3S, collapsed significantly compared to host (HN) of HNΦ. Three dimensional visualization field and histograms also highlighted difference in X, Y, Z scale of both uninfected and infected host cells. The surface topological differences of the lysed host were the target of the study. The current resolution is beyond the visual range for capturing phage particle on the bacterial surface. The AFM images reflected the post infection consequences at the time of lysis not the course of infection which would demonstrate temporal and spatial details of the study. AFM Images of control and HNΦ infected bacteria on Poly-l-lysine mica surface are shown in Fig. 2a–f, control and 3S infected bacteria in Fig. 3a–f.

## Discussion

Nowadays scientists are more interested towards Phage therapy to treat UTI because uropathogenic strains are becoming resistant to antibiotic treatment. The gene for antibiotic resistance is located on mobile genetic elements which can be transmitted to other bacterial species that increase the chances of recurring infection (Foxman and Buxton 2013). Counting on the benefits of phage therapy for uropathogens, main focus of study was to isolate and characterize the lytic phage of uropathogenic *E. coli* strain and study two phages 3S and HNΦ on different parameters comparatively.

In this work two sewage-derived phage HNΦ and 3S were isolated and found lytic on UTI causing *E. coli* strains HN, N.E. respectively as shown in Table 1. Certainly, it is because of receptor compatibility between the phage and bacteria that allows phage to infect bacteria and initiate lytic cycle in the host. Host belonging to different bacterial genera were used to characterize host range of HNΦ and 3S and it became evident that these phages are specific with narrow host range as shown in Table 1. According to Wang and Lin (2001), the key reason for possible interaction between phage and bacteria is dependent on the composition of Lipopolysaccharides (LPS). Mostly bacterial strains consist of LPS having similar composition but some strains also possess different composition of LPS. The phage lytic for all other similar strains become non lytic towards bacteria having different LPS composition, because of incompatibility between surface receptor and phage. The factor that influences difference in LPS composition is the environment of strains from which they were isolated.

The classical experiment of one step growth curve was carried out to differentiate phages on the basis of burst size. It was found that both phages HNΦ and 3S are high titre phages which have latent period of 30 min and burst size for HNΦ was 110 but for 3S it was 42. It can be speculated on the basis of latent period that both phages may belong to T-even series because 30 min duration of latent period is usually found for T-even *E. coli* phages, as calculated from Fig. 1. According to Shao and Wang (2008), phages that are characterized as Short latent periods phages (SLP_s_) have high adsorption rate, shorter latent period and they are able to infect multiple host in the environment, but they have reduced burst size. These findings of one step growth curve support the idea that HNΦ and 3S may be taken as SLP_S_ on the basis of latent period and burst size. The host range of phages was checked on heterologus host i.e. Staphylococcus, Pseudomonas, Shigella and different species of Salmonella and it was found that these phages were non lytic to any of these bacteria. But absence of plaques cannot rule out the possibility of lysogenic state of this phage into any of the host. However, screening of inducing factor was not done in present study. Previous research showed that T phages whose latent period is about 30 min have burst size in the range of 200–300 PFU/infection centre but for HNΦ burst size is 110 PFU/infection centre and for 3S is 42 PFU/infection centre therefore only on the basis of short latent period and small burst size it is concluded that these phages are SLPs. However, further research is needed to determine adsorption rate and its activity against multiple hosts to confirm them as SLP_s_.

In addition to microbiological assays, Atomic Force Microscopy (AFM) was also used to investigate the structural and topological effect of the phage on host cell. Initial experiments were performed on Gelatin and Poly l-Lysine coated mica surface. It was found that images obtained by Poly l-Lysine coated mica surface were clearer than gelatin coated surface. Since Poly l-Lysine provides more even and firm layers than gelatin, therefore, Poly l-Lysine coated surface were used for subsequent experiments. This method gave excellent images in which control bacteria were visualized as regular, solid rods with structural integrity but infected bacteria showed topological changes in the overall cell structure. For phage infected HN host cells central depression is found as shown in Fig. 2d–f, also supported by 3D diagram which shows that cantilever moves to larger distance in Z axis for infected bacteria because cell lost its integrity and became flatten as compared to normal host cells. It showed that DNA of lytic phage after injection in host cell is directed towards the bacterial nucleoid located in the centre of bacterial cell and later propagation, assembly and maturation of phage particles take place at the central region of nucleoid which ends up on the release of progeny particles by bursting of the central region of the host cell. Whereas the lytic activity of 3S phage is shown in Fig. 3d–f which exhibited the exit of the progeny from multiple sites of the host cell disrupting the entire structure of host cell. Histograms of infected host cells (NE and HN) of 3S and HNΦ phages respectively indicated major differences in the 3D scale of the field as shown in Figs. 2f and 3f. The multiple exit sites exhibited in the image actually referring to the site of morphogenesis of the phage in the host cell that is presumably the phage replication and assembly which take place at peripheral region of the nucleoid.

## Conclusion

The evolution of Multidrug-resistant (MDR), Extensively drug-resistant (XDR) and Pandrug-resistant (PDR) bacteria are becoming universal issue overall in medical sciences because the emergence of such type of bacterial strains are causing life threatening diseases. To resolve this situation scientist are looking towards phage therapy to treat such life threatening diseases instead of using antibiotics. Conventional method to rule out efficacy of phages against host cell is time consuming so latest techniques are developing to speed up the whole process of screening of lytic phages which will sort out isolated phage relatively lesser time as a potential tool for phage therapy. Out of these technique AFM is the latest advance technique which facilitate visualizing interaction of phage and bacteria in real time. This technique is used in the research to evaluate potential of isolated phages to lyse bacterial host cells and observe that isolated phages are potential candidate for phage therapy. Details of phage and host interaction in temporal and spatial perspective can be further studied.

## Abbreviations

UTI: urinary tract infections
*E. coli*: *Escherichia coli*
UPEC: uropathogenic *E. coli* strains
PD: pharmacodynamics
PK: pharmacokinetics
AFM: atomic force microscopy
TEM: transmission electron microscopy
BHI: brain heart infusion agar
TSI: triple sugar iron
EMB: eosin methylene blue agar
LB: luria–bertani
PDB: phosphate dilution buffer
HN: isolated *E. coli* strain
HN Φ: isolated phage for HN *E. coli* strain
NE: isolated *E. coli* strain
3S: isolated phage for HN *E. coli* strain
LPS: lipopolysaccharides
SLPs: short latent periods phages
MDR: multidrug-resistant
XDR: extensively drug-resistant
PDR: pandrug-resistant
